# On the Claims of Dr. Morgan and Mr. Hunter to the Theory of Pus as a Secretion

**Published:** 1818-04

**Authors:** 


*For the London Medical and Physical Journal.*

### *On the Claims of Dr. Morgan and Mr. Hunter to the Theory of Pus as a Secretion.*

IN a former Number, Dr. Curry was invited to give the passage in Dr. Morgan's inaugural Theses, containing his theory concerning the formation of pus. As this has not yet appeared, we are obliged to a correspondent for the following extract. But, first, we shall give, in the words of Dr. Morgan, the theory of Dr. William Hunter, published the preceding year.

"Postremo, hac dissertatione ad finem ferè perductâ, mihi volupe erat occasionem habuisse perlegendi quid de hac re nuper scriptum est nempe.

"CELEBERRIMUS G. HUNTERUS 'Pus duplex esse in genere notat. Aliud, dicit, manifestam solidorum rupturam comitari, quale in abscessu communi videmus; aliud super internas cavitatum superficies inflammatione obsessas, absque ulla exulceratione visibili, aut solidorum continuitatis solutione, collectum esse; quod exsudationem inflammatoriam appellat. Et concludit, Puri semper inflammationem præire, aut ab hac illud produci. Primum fluidum esse ponit ex succis qui in vasis distentis contenti fuerunt, et ea parte vasorum, quæ suppuratione soluta aut liquefacta erat, constans. Posterius tantummodo naturam habere seri inspissati autumat. Et Pus in omni vulnere sanescente, e succis solum, non e fluidorum et solidorum miscela constare arbitrarur.*

"'§. III. Quum neque harum ulla, aut alia quæcunque mihi nota sententia de *Puopoiesi*, ad eam quæ jamdudum mihi verisimilis apparuit, plenè accedit, non abs re fore puto eas collegisse, quibus maxima est auctoritas, ut uno intuitu conspicerentur. Has etiam inter se diligenter comparavi, antequam in medium proferre meam ausus essem, ut non opinionum celebriorum ignorantia ab iis dissensisse vel inconsulto nimis aliquid novi, quod minime velim, proposuisse videar. Dum viri autem gravissimi de Puris Confectione inter se discrepant, res postulat ut sedulo perpendatur. In tot sententiarum diversitate meam etiam proponere licet, nempe, PURIS CONFECTIONEM, ŒCONOMIÆ ANIMALIS OPUS ESSE, SECRETIONI MAXIME ANALOGUM, AUT REVERA FLUIDI PECULIARIS SECRETIONEM EX SANGUINE VIVI, FACTAM VIRTUTE VASORUM, EX DEBITO GRADU INFLAMMATIONIS PRÆEUNTIS, HUIC ACCOMMODATORUM.'"

By the above passage, it is evident, that Dr. Hunter did not consider a solution of solids as necessary for the production of pus, having discovered it in parts in which there was no apparent ulceration. Observing further, that it is always an attendant on granulating and healing wounds, he considered pus as of the nature of inspissated serum.

"* London Medical Observations and Inquiries, vol. ii. art. 2.

Dr. Morgan's own opinion follows, viz. that the formation of pus is an operation of the animal economy *very analogous* to secretion, or actually a secretion of a peculiar living fluid from the blood, caused by the vessels being accommodated to that purpose by a certain degree of preceding inflammation.

Dr. M. then proceeds to show the difference between his opinion and that of his predecessors. His difference from Dr. Hunter is expressed with sufficient perspicuity, namely, that pus is not formed from any part of the blood after it has been forced from its vessels, but is an actual secretion, induced by an alteration in the action of those vessels in consequence of inflammation.

"Celeb. vero HUNTERUM cum sententia communi asserere inflammationem puris generationi præire. Hoc mea speciale habet, Pus nempe neque in sanguine neque extra vasa generari, sed in ipsis vasis inflammatis; et vasorum mutationes ab inflammatione inductas, esse causas efficientes, quæ, virtute quadam secretoria, Pus e sanguine eliciunt."

No one would doubt, after this, that Dr. Morgan improved a theory of which Dr. Hunter traced only the more obvious phenomena. But, if we attend to dates, we shall find Dr. W. Hunter, 1764; Dr. Morgan, 1765; and we ought to recollect, that, though Mr. J. Hunter did not publish his opinions till several years after, yet he was an assistant-lecturer to his brother in the year 1759; that is, six years before the date of Dr. Morgan's publications; and also that, though the society, in whose Memoirs Dr. Hunter's paper appears, did not publish their second volume till a year before Dr. Morgan's Theses, yet that the subject had been matter of discussion in London for several years. To prove this, we need only copy the following passage from Mr. Hunter's Treatise on Gonorrhœa.

"It may not be improper (says he,) to give here a short history of the discovery of matter being formed by inflammation without ulceration. In the winter 1749, a child was brought into the room used for dissection, in Covent-Garden, on opening of whose thorax a large quantity of pus was found loose in the cavity, with the surface of the lungs and the pleura furred over with a more solid substance, similar to coagulable lymph. On removing this from those surfaces, they were found entire. This appearance being new to Dr. Hunter, he sent to Mr. Samuel Sharp, desiring his attendance, and to him it also appeared new. Mr. Sharp afterwards, in the year 1750, published his Critical Enquiry, in which he introduced this fact, 'That matter may be formed without a breach of substance;' not mentioning whence he had derived this notion. It was ever after taught by Dr. Hunter in his lectures; we, however, find writers adopting it, without quoting either Mr.

Sharp or Dr. Hunter. So much being known, I was anxious to examine whether the matter in a gonorrhœa was formed in the same way. In the spring of 1753, there was an execution of eight men, two of whom, I knew, had at that time very severe gonorrhœas. Their bodies being procured for this particular purpose, we were very accurate in our examination, but found no ulceration, the two urethras appeared merely a little blood-shot, especially near the glans. This being another new fact ascertained, it could not escape Mr. Gataker, ever attentive to his emolument, who was then attending Dr. Hunter's lectures, and also practising dissection under me. He published soon after, in 1754, a Treatise on this disease, and explained fully, that the matter in a gonorrhœa did not arise from an ulcer, without mentioning how he acquired this knowledge; and it has ever since been adopted in publications on this subject. Since the period mentioned above, I have constantly paid particular attention to this circumstance, and have opened the urethra of many who, at the time of their death, had a gonorrhœa, yet have never found a sore in any; but always observed, that the urethra near the glans was more blood-shot than usual, and that the lacunæ were often filled with matter. I have, indeed, seen an instance of a sore a little within the urethra; but this sore was not produced by any ulceration of the surface, but from an inflammation taking place probably in one of the glands, which produced an abscess in the part, and that abscess opened its way into the urethra. The very same sore opened a way through externally at the frænum, so that there was a new passage for the urine. Indeed, the method of curing a gonorrhœa might have shown that it could not depend upon a venereal ulcer; for there is hardly an instance of a venereal ulcer being cured but by mercury, except by escharotics. We know, however, that most gonorrhœas are curable without mercury; and what is still more, without any medical assistance, which I believe is never the case with a chancre. This fact, of there being no ulcers, was first taught publicly by Dr. Hunter at his lectures in the year 1750, but he did not attempt to account for it."

In the perusal of Dr. M.'s Thesis, three other observations occur: first, that he adopts completely the Boerhaavian theory of inflammation, that is, obstruction in the finer vessels; and from thence shows the necessity of the formation of pus to dislodge the obstructed part, or the substances which induce the obstruction. Secondly, that, when inflammation is confined to a part, he calls it a local, or *topical, fever*, proving from Galen and Celsus that such cases occur, called by the latter, "*transfusio sanguinis in eas venas quæ spiritui accommodatæ sunt;*" evidently mistaking the meaning of the ancients, who supposed that, in a state of health, the arteries contained nothing but air, from which they derived their name. Lastly, we no where meet with the most powerful argument in support of pus as a secreted fluid; namely, that secreting surfaces, or mucous membranes under inflammation, secrete pus, and that pus from this source has all the properties and all the varieties we meet in the pus which is formed on ulcers, depending on the condition of the parts, and the degree of inflammation attending them.

The above are only offered as hints for those who wish to pursue the inquiry further, and we shall be particularly thankful for any information, whether Dr. Morgan spent any part of a winter in London previous to his graduation at Edinburgh.

We may further observe, that, by the passage extracted from Mr. Hunter's Treatise, the only dispute at that time was concerning the nature of pus unconnected with ulceration; and this is all we meet with in Dr. Morgan. But a new question necessarily arises:—If pus does not consist of a *melting down* of the solid matter in ulceration, how is the solid part which is lost by ulceration disposed of? This is readily explained in Mr. Hunter's subsequent writings, and is now pretty generally understood; but Dr. Morgan is entirely silent on that part of the question.

